# Flagellin attenuates experimental sepsis in a macrophage-dependent manner

**DOI:** 10.1186/s13054-019-2408-7

**Published:** 2019-04-03

**Authors:** Xiaoliang Yang, Yibing Yin, Xingxing Yan, Zebo Yu, Yi Liu, Ju Cao

**Affiliations:** 1 Department of Blood Transfusion, The First Affiliated Hospital of Chongqing Medical University, Chongqing, China; 2 Key Laboratory of Diagnostic Medicine designated by the Ministry of Education, Chongqing Medical University, Chongqing, China; 3 Department of Laboratory Medicine, The First Affiliated Hospital of Chongqing Medical University, No. 1 Youyi Road, Yuzhong District, Chongqing, 400016 China; 4 Department of Intensive Care Unit, The Second Affiliated Hospital of Chongqing Medical University, Chongqing, China

**Keywords:** Flagellin, Sepsis, Macrophages, Monocytes, TLR5

## Abstract

**Background:**

Sepsis is the leading cause of death among critically ill patients, and no specific therapeutic agent is currently approved for the treatment of sepsis.

**Methods:**

We assessed the effects of flagellin administration on survival, bacterial burden, and tissue injury after sepsis. In addition, we examined the effects on phagocytosis and bacterial killing in monocytes/macrophages.

**Results:**

Therapeutic administration of flagellin increased bacterial clearance, decreased organ inflammation and injury, and reduced immune cell apoptosis after experimental sepsis, in a Toll-like receptor 5 (TLR5)–dependent manner. Macrophages, but not neutrophils, mediated the beneficial effects of flagellin on experimental sepsis, and flagellin induced macrophage polarization into M1 in septic mice. Flagellin treatment could directly enhance phagocytosis and bacterial killing of macrophages, but not neutrophils. Subsequent studies demonstrated that flagellin could promote phagosome formation and increase reactive oxygen species (ROS) levels in macrophages. Finally, we found that the expression of TLR5 was significantly elevated on the surface of circulating monocytes, but not neutrophils, from patients with sepsis. Higher expression levels of TLR5 on monocytes were associated with increased mortality, documented bacteremia, and higher Sequential Organ Failure Assessment scores of the septic patients. Moreover, flagellin treatment rescued the impaired phagocytosis and bacterial killing ability of monocytes/macrophages from patients who died of sepsis.

**Conclusions:**

These novel findings not only established the potential value of application of flagellin as an immunoadjuvant in treating sepsis, but also provided new insights into targeted therapeutic strategy on the basis of monocyte TLR5 expression in septic patients.

**Electronic supplementary material:**

The online version of this article (10.1186/s13054-019-2408-7) contains supplementary material, which is available to authorized users.

## Background

Sepsis is defined as life-threatening acute organ dysfunction secondary to infection in the body and bloodstream that most commonly originates in the lung, urinary tract, and abdomen [1, 2]. Sepsis progresses when the initial host response fails to control the infection, resulting in widespread inflammation and multiple organ injury [3]. Sepsis-induced mortality is closely associated with the failure to eradicate invading pathogens [4]. In a post-mortem study of 235 patients in surgical intensive care who were admitted with sepsis, about 80% of patients had unresolved septic foci at death [5]. Therefore, new strategies to treat sepsis should boost host immunity, thereby leading to a more rapid resolution of the infection and prevention of death.

Toll-like receptors (TLRs) are central for host defense against pathogenic microorganisms by recognizing conserved motifs in pathogens termed pathogen-associated molecular patterns [6]. TLR5 is the receptor for extracellular flagellin, which is a component of motile bacteria [7]. Clinical data have shown that the expression of TLR5 on monocytes predicted systemic inflammatory response syndrome (SIRS) [8]. Animal data have demonstrated that TLR5 agonist flagellin restored antibiotic-impaired innate immune defenses and restricted colonization with vancomycin-resistant *Enterococcus* (VRE) [9]. However, whether TLR5 plays a role in controlling infection during sepsis has yet to be addressed. In this study, we investigated the role of flagellin-induced TLR5 activation in controlling the infection during sepsis using the cecal ligation and puncture (CLP) model of abdominal sepsis in mice. We also measured TLR5 expression levels in septic patients and analyzed their relationship with clinical phenotypes.

## Methods

### Patient and healthy control demographics

Patients who met the clinical criteria for Sepsis-3 were screened for eligibility within the first 24 h after they were admitted to the Department of Infectious Diseases of The First Affiliated Hospital of Chongqing Medical University or the Intensive Care Unit of The Second Affiliated Hospital of Chongqing Medical University between January 2017 and February 2018 [1, 10]. A total of 53 septic adult patients (Additional file 1: Table S1) were enrolled. Patients were included if they had known or suspected infection plus an increase in the Sequential [Sepsis-related] Organ Failure Assessment (SOFA) score of 2 or more points for organ dysfunction. Patients who are pregnant or breast-feeding; patients with malignancy, organ transplantation, chronic viral infections (hepatitis, HIV), liver cirrhosis, chronic kidney insufficiency, and autoimmune diseases; and patients using immunosuppressive medication were excluded from the study. Twenty-three non-septic patients but in critical conditions of trauma injury (poly-trauma or cerebral trauma) were recruited as controls. The clinical data, such as Acute Physiology and Chronic Health Evaluation II (APACHE II) score, SOFA score, causes of sepsis, microbial culture result, length of intensive care unit stay, and mortality, were recorded. There was no difference in end-stage renal failure between septic patients and non-septic patients. Healthy control samples were obtained from 37 healthy donors with no medical problems in the medical examination center of The First Affiliated Hospital of Chongqing Medical University. Nine milliliters of venous peripheral ethylenediaminetetraacetic acid (EDTA) blood was collected at the time of enrollment, and blood samples were also obtained from 9 patients with sepsis within 1 h of death. Aliquots of whole blood were processed immediately for peripheral blood mononuclear cell (PBMC) isolation. PBMCs were prepared by centrifugation of blood using a density gradient (Ficoll-Paque Plus; GE Healthcare Life Sciences). This protocol was approved by the Clinical Research Ethics Committee of Chongqing Medical University, and informed consent was obtained from all participants according to the Declaration of Helsinki.

### Sepsis model

Pathogen-free 6–8-week-old female C57BL/6 mice were obtained from and raised at Chongqing Medical University. Polymicrobial sepsis was provoked by CLP as described in our previous studies [11, 12]. C57BL/6 mice were anesthetized with a mixture of xylazine (4.5 mg/kg) and ketamine (90 mg/kg) intraperitoneally (i.p.). CLP was performed by making a midline incision ~ 2.5 cm in length to expose the cecum. A 3-0 silk ligature was placed at the base of the cecum without causing bowel obstruction. The cecum was then punctured twice with a 21-gauge needle (lethal CLP) or a 26-gauge needle (sublethal CLP). The cecum was then placed back in the peritoneal cavity, and the incision was closed with surgical staples. Sham-operated (control) animals underwent identical laparotomy, the cecum was exposed but not ligated or punctured and was then replaced in the peritoneal cavity. Mice received saline (5 ml per 100 g body weight) subcutaneously for resuscitation, and no antibiotic treatment was used. Survival was monitored twice daily for 14 days. For the *Escherichia coli* model, 5 × 10^8^ *E*. *coli* was injected intraperitoneally. The animal experiments were approved by the local Animal Care and Use Committee.

### Flagellin

For animal experiments, TLR5 ligand flagellin (InVivoGen), derived from *Salmonella typhimurium*, was used. Flagellin did not contain detectable lipopolysaccharide (LPS), as determined by the Limulus amoebocyte lyase assay (sensitivity limit 12 pg/ml; BioWhittaker Inc., Walkersville, MD). Flagellin was injected i.p. at 2–10 μg/injection at 2–8 h after CLP, and PBS was delivered in a similar fashion as control solutions.

### Treatment with anti-TLR5 antibody and recombinant mouse TLR5 Fc Chimera

TLR5 neutralization was performed by i.p. administration of 50 μg of rat anti-mouse TLR5 monoclonal antibody (InVivoGen, clone: Q23D11) at 2 h before CLP. The neutralization activity has been determined for its ability to inhibit flagellin-induced NF-kB activation in HEK-293 cells transfected to express mouse TLR5. Rat IgG2a was used as isotypical IgG antibody. In some experiments, TLR5 inhibition was also performed by i.p. administration of 50 μg recombinant mouse TLR5 Fc Chimera (R&D systems) at 2 h before CLP. The inhibition effect has been confirmed for its ability to inhibit flagellin-induced IL-8 secretion in HT29 human colon adenocarcinoma cells.

### Statistics

Data were expressed as scatter dot plots with medians unless otherwise specified. Comparisons between groups were tested using Student’s *t* test, Mann–Whitney *U* test, or Kruskal–Wallis test followed by Dunn’s multiple comparisons post-test as appropriate. Correlations were tested by Spearman’s rank correlation test. For survival studies, Kaplan–Meier analyses followed by log-rank tests were performed. All analyses were done using GraphPad Prism version 5.01 (GraphPad Software, San Diego, CA). *p* values less than 0.05 were considered statistically significant.

Additional details on the methods are available in the online supplement.

## Results

### Flagellin protected against abdominal sepsis via TLR5

**Fig. 1:**
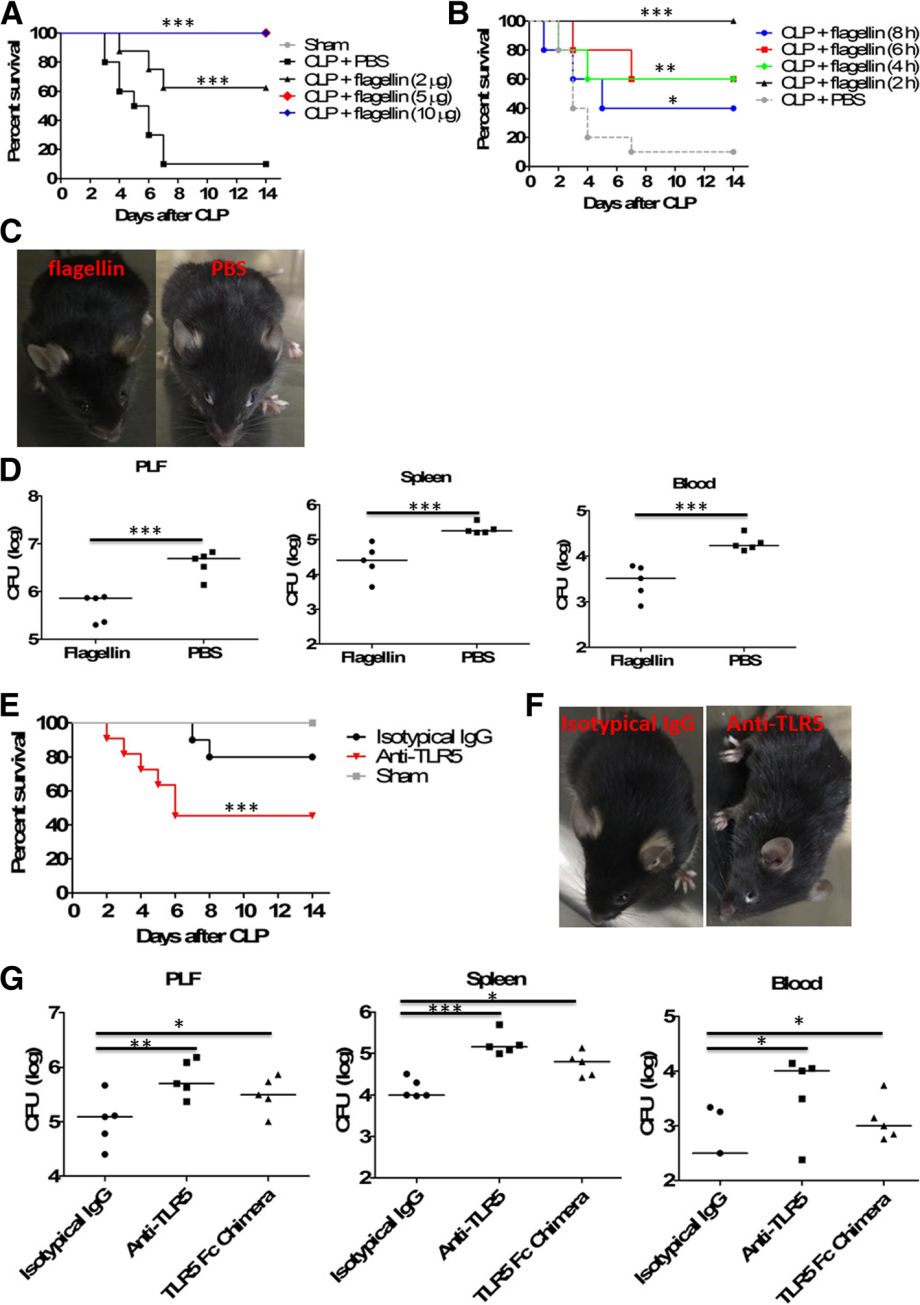
Therapeutic effects of flagellin in experimental sepsis. **a** Various doses of flagellin (0, 2, 5, 10 μg) were injected intraperitoneally into cecal ligation and puncture (CLP)–induced septic mice at 2 h after lethal CLP using 21-gauge needle, and survival was monitored (*n* = 16 per group). Comparison between groups was done by Kaplan–Meier analysis followed by log-rank tests. ***p < 0.001 when compared with mice treated with phosphate-buffered saline (PBS) control. **b** CLP mice were given intraperitoneally 5 μg of flagellin at 2, 4, 6, and 8 h after CLP, and survival was monitored (*n* = 16 per group). Comparison between groups was done by Kaplan–Meier analysis followed by log-rank tests.*p < 0.05, **p < 0.01, and ***p < 0.001 when compared with mice treated with PBS control. **c** Clinical appearance of flagellin-treated (5 μg) and control mice at 24 h after CLP. **d** Bacterial counts in peritoneal lavage fluid (PLF), spleens, or blood from mice (*n* = 5 per group) treated with or without flagellin (5 μg) at 24 h after CLP. Horizontal bars represent median values, and dots represent individual mice. ***p < 0.001when compared between groups (denoted by horizontal bracket; Mann–Whitney *U* test). **e** CLP mice (*n* = 20 per group) were given intraperitoneally 50 μg of anti-TLR5-neutralizing monoclonal antibodies at 2 h before CLP, followed by flagellin (5 μg) treatment at 2 h after CLP, and survival was then monitored. Comparison between groups was done by Kaplan–Meier analysis followed by log-rank tests. ***p < 0.001 when compared with mice treated with isotypical IgG. **f** Clinical appearance of flagellin-treated (5 μg) mice with or without TLR5 neutralization at 24 h after CLP. **g** Bacterial counts in PLF, spleens, or blood from flagellin-treated (5 μg) mice (*n* = 5 per group) with or without TLR5 blockade using anti-TLR5 antibodies (50 μg) or TLR5 Fc Chimera (50 μg) at 24 h after CLP. *p < 0.05, **p < 0.01, and ***p < 0.001 when compared with control mice

To determine whether flagellin was sufficient to improve mortality in lethal sepsis, we examined the effects of flagellin administration using a mouse model of CLP-induced lethal sepsis. Fourteen days after CLP, untreated C57BL/6 mice had over 95% mortality. However, administration of flagellin 2 h after CLP dramatically increased the survival rate in a dose-dependent manner, and injection of flagellin (5 or 10 μg) resulted in 100% survival in mice (Fig. 1a). In terms of injection time, survival was greatly improved when flagellin (5 μg) was injected 2 (100% survival), 4 (60% survival), or 6 (60% survival) hours post-CLP (Fig. 1b). When flagellin was injected 8 h post-CLP, the therapeutic effect was still shown (40% survival) compared to the PBS control (10% survival). Given these results, our subsequent experiments were performed in CLP mice, using flagellin (5 μg) 2 h after CLP. In line with an increased mortality rate, control mice presented with lethargy and piloerection as soon as 24 h after CLP, which are clinical signs of severe sepsis (Fig. 1c), and we also studied bacterial loads in peritoneal lavage fluids (PLF), spleen, and blood after 24 h of CLP showing markedly reduced bacterial numbers in flagellin-treated animals (Fig. 1d).

The effects of flagellin in another model of sepsis were also evaluated. Therapeutic administration of flagellin at 2 h after infection significantly enhanced survival in mice inoculated with *E*. *coli* compared with saline-treated controls (Additional file 1: Figure S1A). Flagellin treatment also significantly reduced bacterial burdens in the PLF and blood after *E*. *coli* infection compared with saline-treated mice (Additional file 1: Figure S1B).

We next asked whether the antiseptic activity of flagellin acts via its reported receptor, TLR5. In mice pretreated with anti-TLR5-neutralizing monoclonal antibodies, the protection effect elicited by flagellin treatment was significantly impaired compared with mice treated with control IgG post-CLP (Fig. 1e). Neutralization with TLR5 using monoclonal antibodies against TLR5 led to lethargy and piloerection in flagellin-treated mice at 24 h after CLP (Fig. 1f), and it significantly impaired bacterial clearance in flagellin-treated mice from the peritoneum, spleen, and blood (Fig. 1g). Pretreatment with recombinant mouse TLR5 Fc chimera also significantly impaired the improvement of bacterial clearance in flagellin-treated mice (Fig. 1g). These results indicated that flagellin acted specifically through TLR5 to protect against experimental sepsis.

### Flagellin inhibited vital organ inflammation and injury as well as immune cell apoptosis

**Fig. 2:**
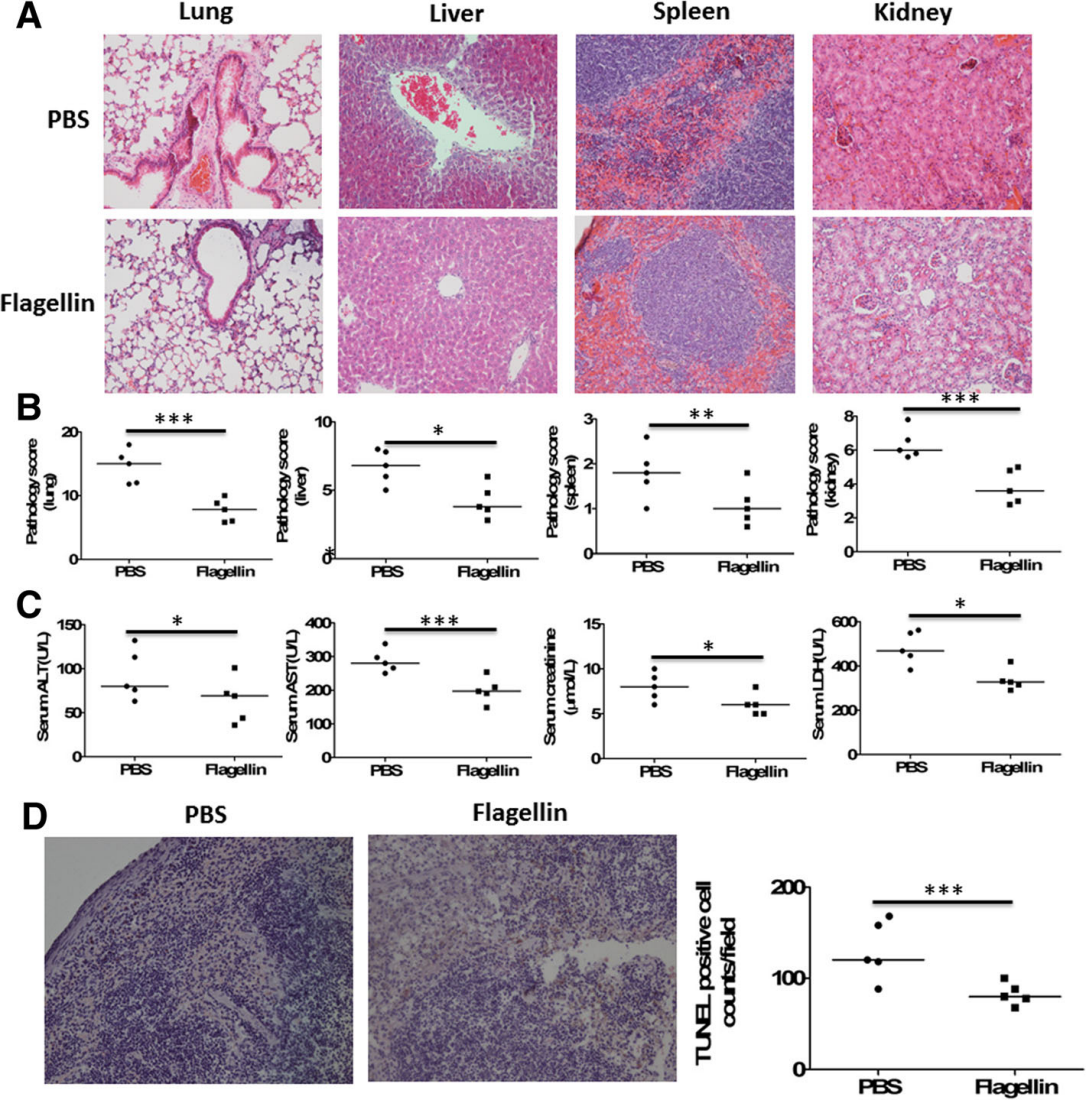
Effect of acetylated flagellin on cecal ligation and puncture (CLP)–induced inflammation and injury of vital organs and immune cell apoptosis. **a** Representative examples of hematoxylin and eosin (H&E)–stained lung, liver, spleen, and kidney tissues from mice (*n* = 5 per group) treated with or without flagellin (5 μg) at 24 h after lethal CLP. **b** Histological scores for lung, liver, spleen, and kidney tissues from mice (*n* = 5 per group) treated with or without flagellin (5 μg) at 24 h after CLP. *p < 0.05, **p < 0.01, and ***p < 0.001 when compared between groups (denoted by the horizontal bracket; Mann–Whitney *U* test). **c** Serological markers of organ injury including alanine aminotransferase (ALT), aspartate aminotransferase (AST), lactate dehydrogenase (LDH), and creatinine in mice (*n* = 5 per group) treated with or without flagellin (5 μg) at 24 h after CLP. *p < 0.05 and ***p < 0.001when compared between groups (denoted by the horizontal bracket; Mann–Whitney *U* test). **d** The spleen tissues from mice (*n* = 5 per group) treated with or without flagellin (5 μg) at 24 h after CLP were subjected to DNA fragmentation analysis (terminal deoxynucleotidyltransferase dUTP nick end labeling [TUNEL]). Representative examples were shown, and TUNEL-positive cells were counted. ***p < 0.001when compared between groups (denoted by the horizontal bracket; Mann–Whitney *U* test)

The survival of flagellin-treated mice after CLP was associated with improvement of organ dysfunction (Fig. 2). At 24 h after lethal CLP, tissue inflammation and injury of the lung, liver, spleen, and kidney was reduced by the administration of flagellin (Fig. 2a), which was reflected by significantly lower pathology scores compared with PBS-treated mice (Fig. 2b). Serum concentrations of alanine transaminase (ALT) and aspartate transaminase (AST), creatinine, and lactate dehydrogenase (LDH), markers of the liver, kidney, and general cellular dysfunction, respectively, were also significantly decreased in flagellin-treated mice (Fig. 2c). Furthermore, CLP caused splenic cell apoptosis, which was inhibited by the administration of flagellin (Fig. 2d).

### Flagellin treatment repressed cytokine and chemokine response of septic mice

In line with the observation of improved bacterial clearance and reduced inflammatory response in organs, flagellin-treated animals displayed significantly reduced levels of IL-1β, TNF-α, IL-6, IL-10, and CXCL1 in the PLF and blood at 48 h after CLP when compared with PBS-treated controls (Additional file 1: Figure S2).

### Flagellin-induced protection in sepsis was macrophage dependent

**Fig. 3:**
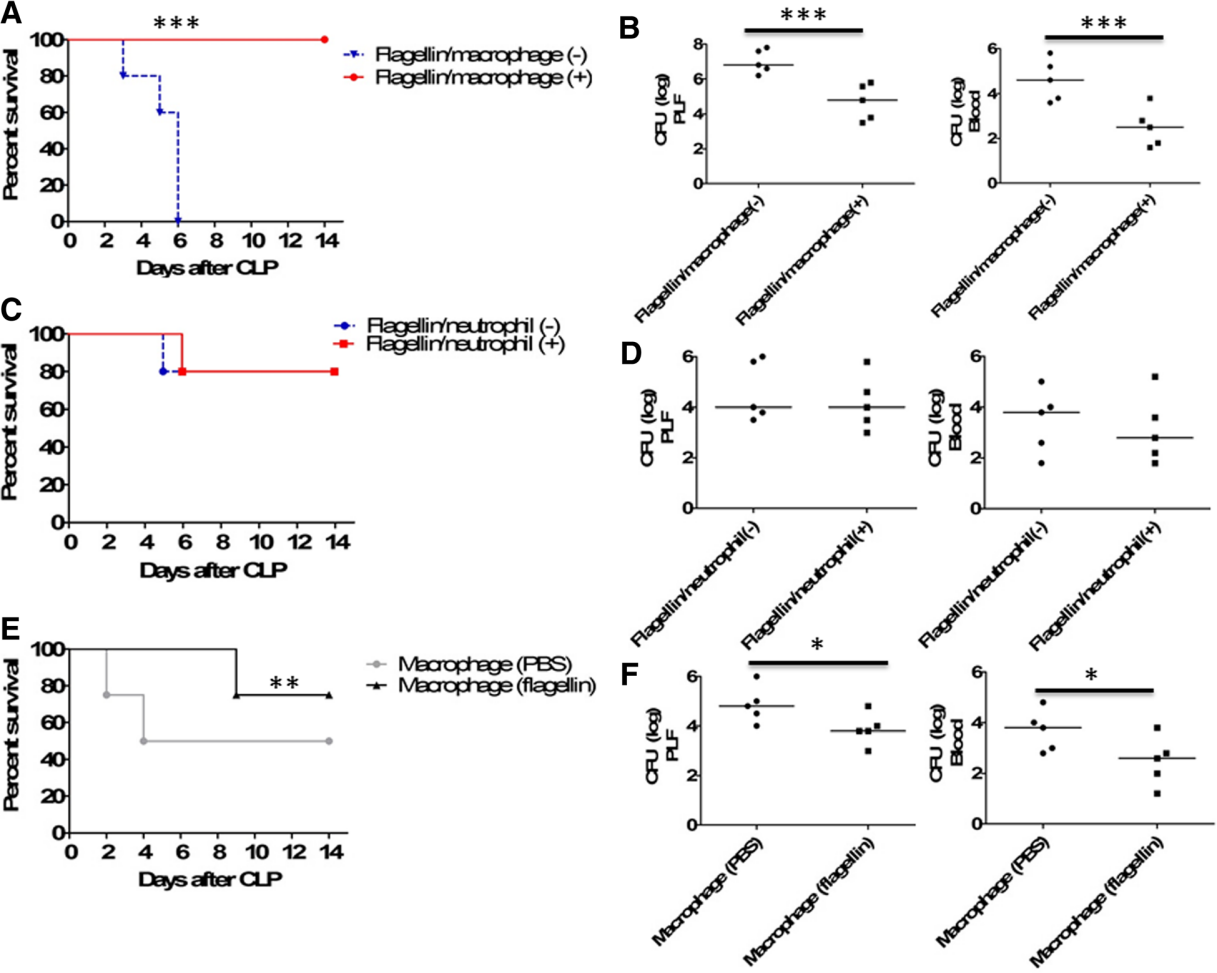
Macrophages were required for flagellin-mediated beneficial effects on cecal ligation and puncture (CLP)–induced sepsis. **a** Mortality after macrophage depletion by clodronate liposomes and subsequent treatment with flagellin (5 μg) or PBS control in mice (*n* = 20) after sublethal CLP using a 26-gauge needle. Comparison between groups was done by Kaplan–Meier analysis followed by log-rank tests. ***p < 0.001 when compared with wild-type mice treated with PBS liposomes as a control. **b** Bacterial counts in PLF and blood from flagellin-treated mice (*n* = 5 per group) with or without macrophage depletion 24 h after CLP. ***p < 0.001when compared between groups (denoted by the horizontal bracket; Mann–Whitney *U* test). **c** Mortality after neutrophil depletion by anti-RB6-8C5 monoclonal antibodies and subsequent treatment with flagellin (5 μg) or PBS control in mice (*n* = 20) after CLP. **d** Bacterial counts in PLF and blood from flagellin-treated mice (*n* = 5 per group) with or without neutrophil depletion 24 h after CLP. **e** Survival after transfer of flagellin-treated macrophages in mice (*n* = 20) with CLP-induced sepsis. Comparison between groups was done by Kaplan–Meier analysis followed by log-rank tests. **p < 0.01 when compared with mice treated with PBS-treated macrophages. **f** Bacterial counts in PLF and blood from mice (*n* = 5) transferred with flagellin-treated or PBS-treated macrophages. *p < 0.05 when compared between groups (denoted by the horizontal bracket; Mann–Whitney *U* test)

Because phagocytes are important for bacterial clearance during sepsis [4, 11, 12], we investigated whether macrophage and/or neutrophil might mediate the beneficial effect of flagellin on sepsis. We first evaluated the importance of macrophages and neutrophils in the survival and bacterial clearance of flagellin-treated septic mice using cell depletion in a sublethal model of sepsis. Depleting macrophages dramatically impaired the survival (Fig. 3a) and bacterial clearance (Fig. 3b) of septic mice treated with flagellin. In contrast, there was no significant change in the survival (Fig. 3c) and bacterial clearance (Fig. 3d) of flagellin-treated septic mice after the depletion of neutrophils. These results suggest that macrophages may associate with the beneficial effect of flagellin in sepsis.

Second, we determined the role of macrophages in flagellin-treated sepsis using adoptive cell transfer. The survival of septic mice receiving flagellin-treated macrophages was significantly higher than that of septic mice receiving saline-treated macrophages (Fig. 3e), and adoptive transfer of flagellin-treated macrophages significantly increased bacterial clearance compared with saline-treated macrophages in septic mice (Fig. 3f). These results suggest that the improved outcomes after flagellin treatment of sepsis are mediated mainly by macrophages.

### Flagellin induced macrophages to M1 polarization in septic mice

**Fig. 4:**
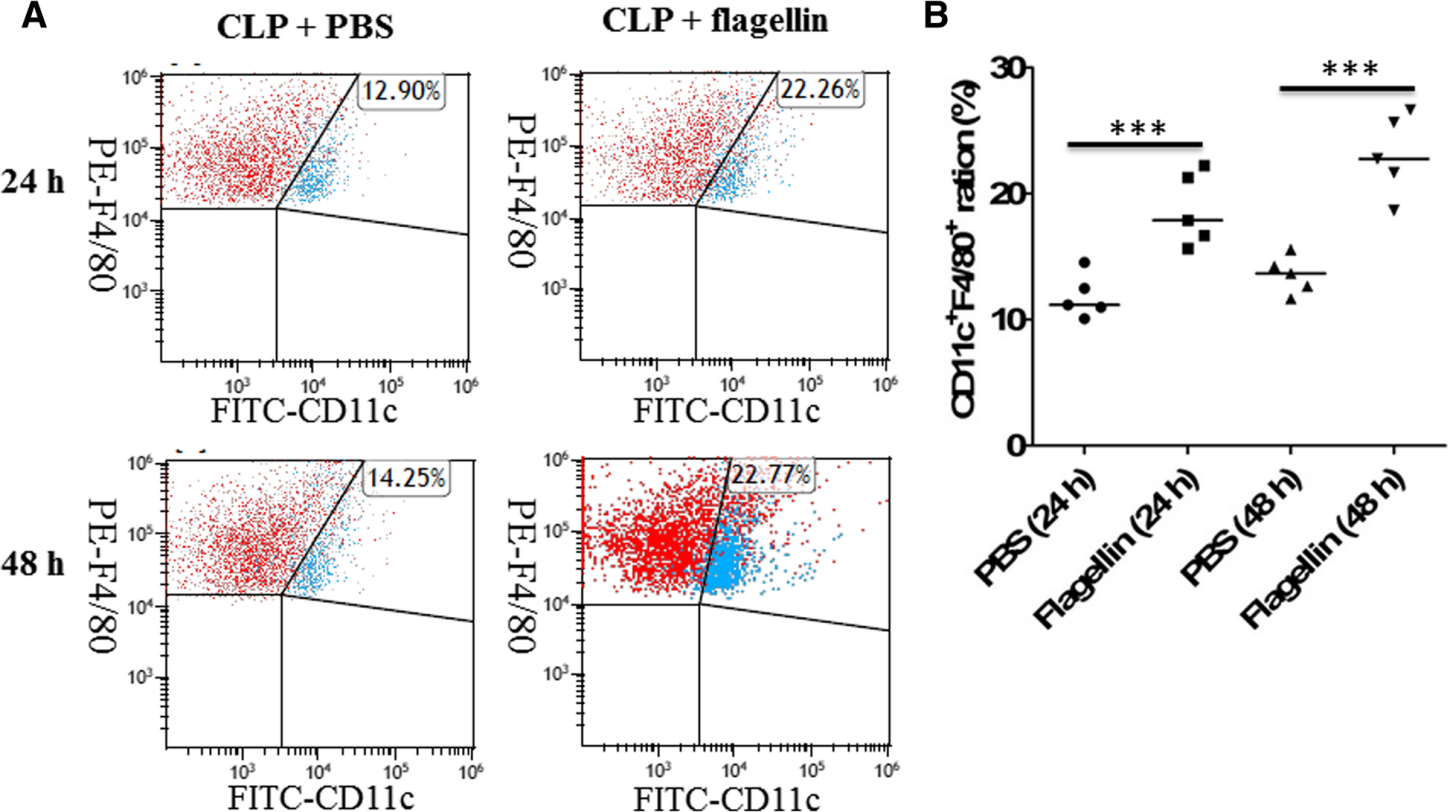
Impacts of flagellin on macrophage M1 polarization in septic mice. **a** Representative dot plots showed an increased percentage of M1 macrophages in PLF from mice (*n* = 5 per group) treated with or without flagellin (5 μg) at 24 or 48 h after CLP. **b** The percentage of CD11c^+^F4/80^+^ macrophages (M1) in PLF from mice (*n* = 5 per group) treated with or without flagellin (5 μg) at 24 or 48 h after CLP. ***p < 0.001 when compared between groups (denoted by the horizontal bracket; Kruskal–Wallis test followed by Dunn’s multiple comparisons post-test)

Classification of macrophage recognizes polarization into two distinct phenotypes, named as proinflammatory (M1) or anti-inflammatory (M2). We further identified whether flagellin treatment induced macrophage polarization in CLP-induced mice. Polarization of macrophage to M1/M2 was determined by CD11c or CD206. Our results exhibited that the numbers of CD11c (M1 marker)-positive cells in septic mice treated with flagellin were significantly higher than those in septic mice treated with PBS control at 24 or 48 h after CLP (Fig. 4a, b). However, there was no significant difference between flagellin- and PBS-treated mice in the positive cells of CD206 (M2 marker) after CLP (data not shown).

### Flagellin directly enhanced antibacterial functions of macrophages

**Fig. 5:**
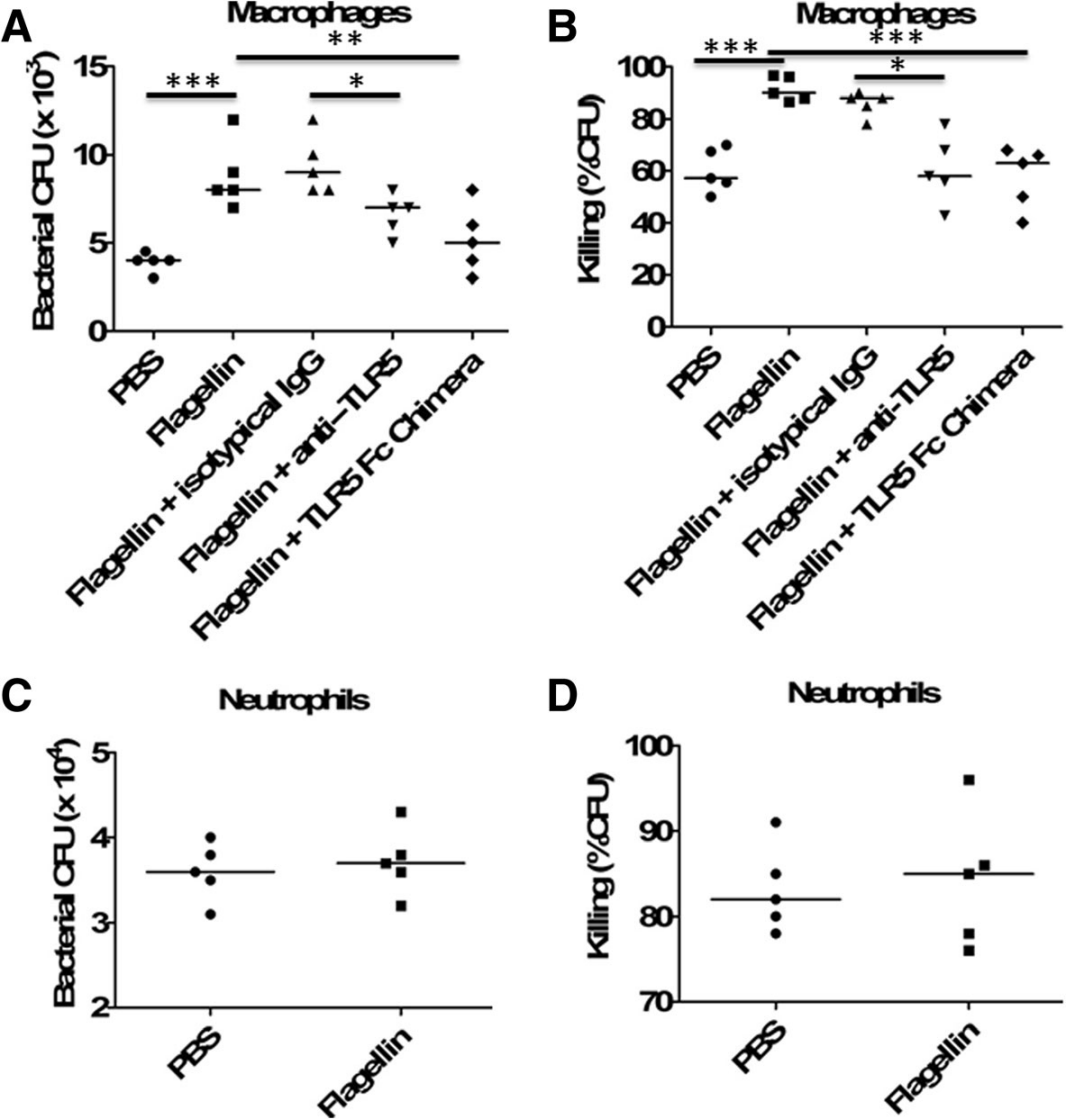
Flagellin enhanced bacterial phagocytosis and killing by macrophages. **a**, **b** Peritoneal macrophages from C57BL/6 mice (*n* = 5 per group) were pretreated with flagellin (100 ng/ml) in the presence or absence of anti-TLR5 antibodies (1 μg/ml) or TLR5 Fc Chimera (1 μg/ml) for 24 h, and then infected with *E*. *coli* (multiplicity of infection, 10) for 30 min. Cells were then washed in PBS and resuspended for 30 min in medium containing 100 μg/ml tobramycin to remove extracellular bacteria. Cells were lysed in PBS containing 0.1% Triton 100 for assessment of **a** phagocytosis (*t* = 0), and additional samples were incubated for 2 additional hours (*t* = 2 h) to assess **b** bacterial killing. Intracellular killing (*t* = 2 h) was determined as described in the “Methods” section. *p < 0.05, **p < 0.01, and ***p < 0.001 when compared between groups (denoted by the horizontal bracket; Kruskal–Wallis test followed by Dunn’s multiple comparisons post-test). **c**, **d** Peritoneal mouse neutrophils were pretreated with or without flagellin (100 ng/ml) for 12 h and then infected with *E. coli* (multiplicity of infection, 100) for 30 min. Cells were then washed in PBS and resuspended for 30 min in medium containing 100 μg/ml tobramycin to remove extracellular bacteria. Cells were lysed in PBS containing 0.1% Triton 100 for assessment of **c** phagocytosis (*t* = 0), and additional samples were incubated for 1 additional hour (*t* = 1 h) to assess **d** bacterial killing

We next attempted to characterize the direct effects of flagellin on macrophages. We observed a significant increase in macrophage phagocytosis of *Escherichia coli* after flagellin stimulation (Fig. 5a), and there was a significant increase in bacterial killing from flagellin-stimulated macrophages (Fig. 5b). Furthermore, blocking TLR5 using anti-TLR5-neutralizing antibodies or recombinant TLR5 Fc chimera could significantly decrease bacterial killing and phagocytosis by flagellin-stimulated macrophages, suggesting that flagellin acted through TLR5 to enhance antibacterial functions of macrophages. However, flagellin did not impact neutrophil-mediated bacterial phagocytosis (Fig. 5c) and killing (Fig. 5d).

### Flagellin promoted phagosome formation and increased reactive oxygen species (ROS) levels in macrophages

**Fig. 6:**
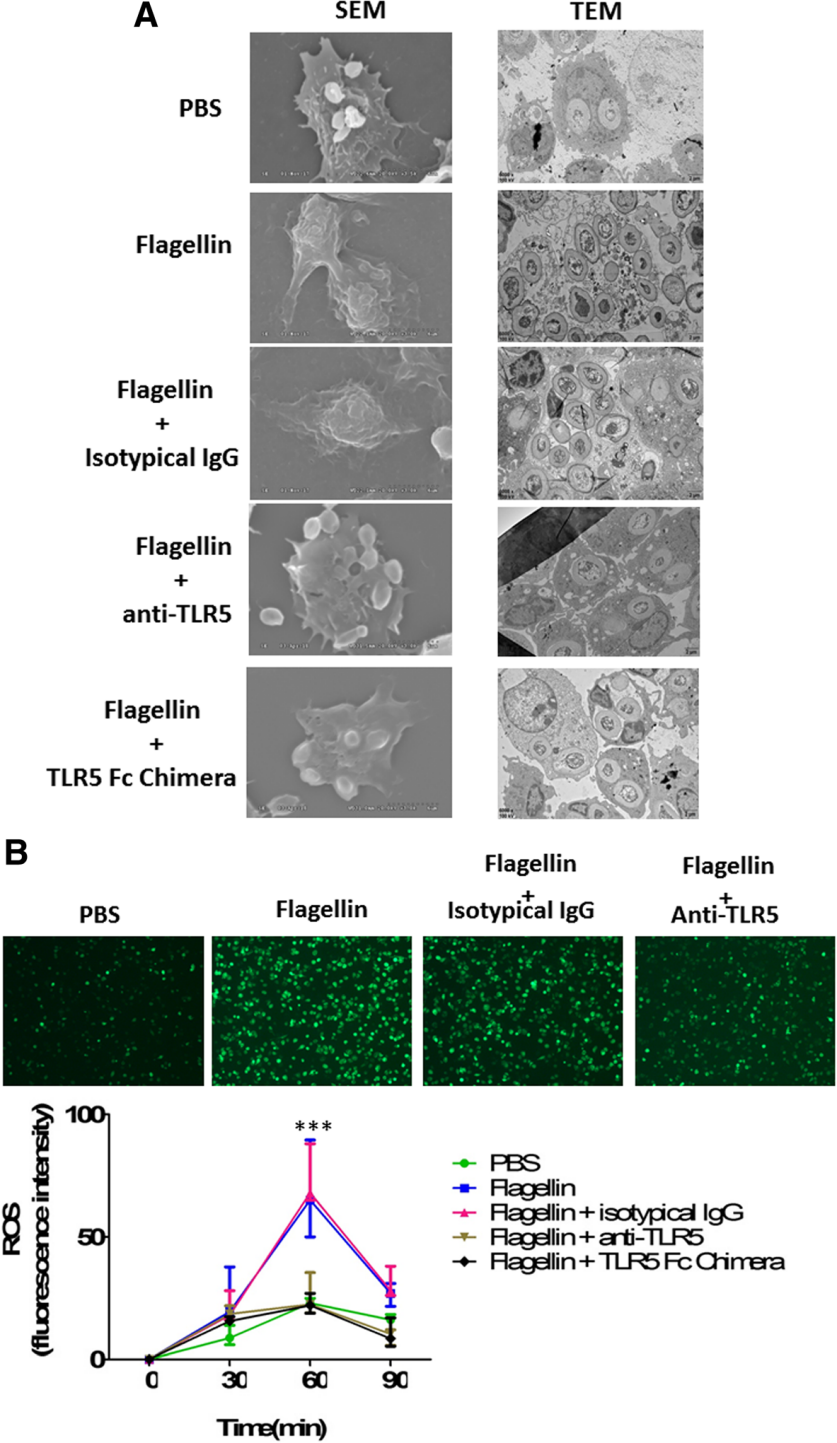
Flagellin promoted phagosome formation and increased reactive oxygen species (ROS) levels in macrophages. **a** Microscopy analysis of zymosan phagocytosis by untreated and flagellin-treated peritoneal macrophages. Peritoneal macrophages from C57BL/6 mice (*n* = 5 per group) were pretreated with flagellin (100 ng/ml) in the presence or absence of anti-TLR5 antibodies (1 μg/ml) or TLR5 Fc Chimera (1 μg/ml) for 24 h, and then challenged with zymosan (20 particles/M_) for 30 min at 37 °C. Scanning electron microscopy showed that zymosan particles were no longer seen on the cell surface of flagellin-treated macrophages, because most have been internalized by macrophages (TEM data). Moreover, TEM showed that fewer zymosan particles were internalized in flagellin-treated macrophages after TLR5 blockade using anti-TLR5 antibodies or TLR5 Fc Chimera when compared with macrophages treated with flagellin alone. **b** Peritoneal macrophages from C57BL/6 mice (*n* = 5 per group) were pretreated with flagellin (100 ng/ml) in the presence or absence of anti-TLR5 antibodies (1 μg/ml) or TLR5 Fc Chimera (1 μg/ml) for 24 h, and then were incubated with heat-inactivated *E. coli*, and ROS levels were assayed at the indicated time points. Representative examples were shown for the production of ROS at 60 min, and the data were also shown as the mean ± standard deviation (SD) and were compared to respective control group at each time point by Student’s *t* test. ***p < 0.001 when compared with control macrophages

We next attempted to investigate whether flagellin-mediated increase of phagocytosis was associated with an increase of phagosome formation in macrophages. Macrophages were stimulated for 48 h with flagellin and then challenged with zymosan. Scanning electron microscopy showed that, by 30 min, no zymosan particle was detected on the surface of flagellin-treated macrophages, and TEM showed that zymosan particles were all intracellular in flagellin-treated macrophages. By contrast, in saline-treated macrophages, many zymosan particles were observed on the cell surface in phagocytic cups, in which distal margins remained open. Furthermore, blocking TLR5 using anti-TLR5-neutralizing antibodies or recombinant TLR5 Fc chimera could inhibit flagellin-mediated phagosome formation in macrophages (Fig. 6a).

One of the key mechanisms by which macrophages kill bacteria involves production of ROS [13]. We observed that flagellin-stimulated macrophages generated significantly more reactive oxygen species in response to *E. coli* than saline-treated macrophages, and blocking TLR5 using anti-TLR5-neutralizing antibodies or recombinant TLR5 Fc chimera could significantly decrease flagellin-induced ROS production in macrophages (Fig. 6b).

### Elevated TLR5 expression on the surface of human peripheral monocytes was associated with severity of sepsis

**Fig. 7:**
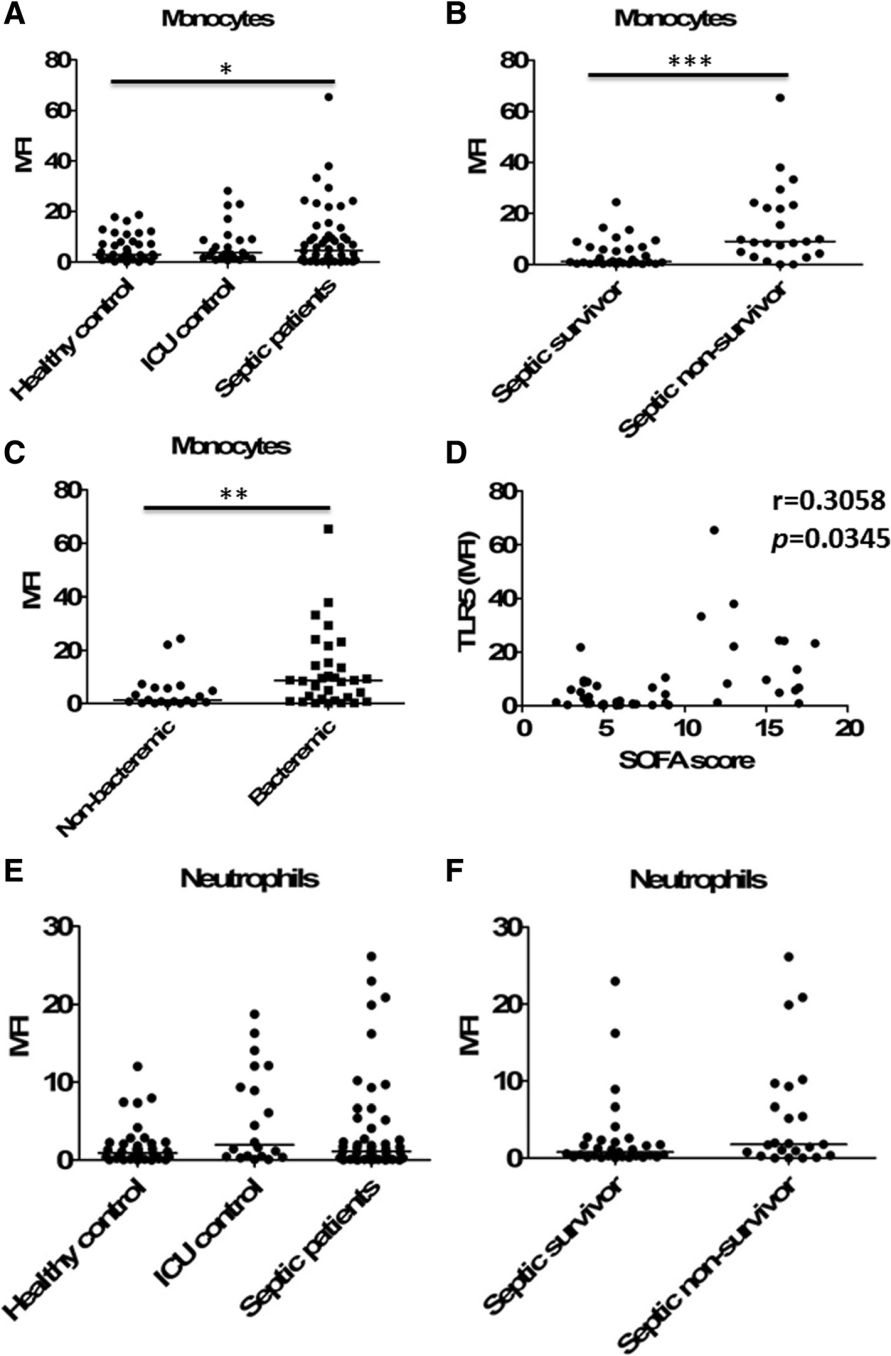
Increased expression of TLR5 on circulating monocytes was related to poorer outcomes of septic patients. **a** The expression of TLR5 on the surface of peripheral blood monocytes from the studied subjects. *n* = 37 for healthy controls, *n* = 23 for ICU controls, and *n* = 53 for sepsis patients. The expression of TLR5 was shown as MFI subtracting corresponding isotypic controls and with scatter plots showing the median. *p < 0.05 when compared between groups (denoted by the horizontal bracket; Mann–Whitney *U* test). **b** The expression of TLR5 on the surface of peripheral blood monocytes from the septic survivors (*n* = 30) and the septic non-survivors (*n* = 23). Horizontal bars represent median values, and dots represent individual participants. ***p < 0.001 when compared between groups (denoted by the horizontal bracket; Mann–Whitney *U* test). **c** The expression of TLR5 on the surface of peripheral blood monocytes from the septic patients with (*n* = 33) or without (*n* = 20) documented bacteremia. **p < 0.01 when compared between groups (denoted by the horizontal bracket; Mann–Whitney *U* test). **d** Correlation of TLR5 expression levels on the surface of peripheral blood monocytes with Sequential Organ Failure Assessment (SOFA) scores in the septic patients (Spearman’s rank correlation test). **e** The expression of TLR5 on the surface of peripheral blood neutrophils from the studied subjects. **f** The expression of TLR5 on the surface of peripheral blood monocytes from the septic survivors (*n* = 30) and the septic non-survivors (*n* = 23)

We further studied the clinical implications of these data. Therefore, 53 septic patients (including 30 survivors and 23 non-survivors), 23 ICU non-septic controls and 37 healthy controls were enrolled (Additional file 1: Table S1 and Table S2), and the expression levels of TLR5 were measured in peripheral blood monocytes using flow cytometry. Compared to those in healthy controls, TLR5 surface expression levels were significantly increased in septic patients. In contrast, ICU non-septic controls showed similar TLR5 expression levels on monocytes with healthy controls (Fig. 7a and Additional file 1: Figure S3). No difference was observed according to the site of infection or microorganism (21 Gram-positive infections, 27 Gram-negative infections, and 5 miscellaneous infections). Furthermore, the septic non-survivors had significantly increased TLR5 expression levels when compared to the septic survivors (Fig. 7b). Higher TLR5 expression levels were associated with documented bacteremia (Fig. 7c) and higher SOFA scores (Fig. 7d) of the septic patients on the day of study enrollment.

In addition, we observed that TLR5 expression levels on the surface of circulating neutrophils from septic patients were not significantly different from those on neutrophils from healthy or ICU non-septic controls (Fig. 7e and Additional file 1: Figure S3), and the septic non-survivors had similar TLR5 expression levels on neutrophils when compared to the septic survivors (Fig. 7f).

### Flagellin enhanced bacterial phagocytosis and killing in human monocytes/macrophages

**Fig. 8:**
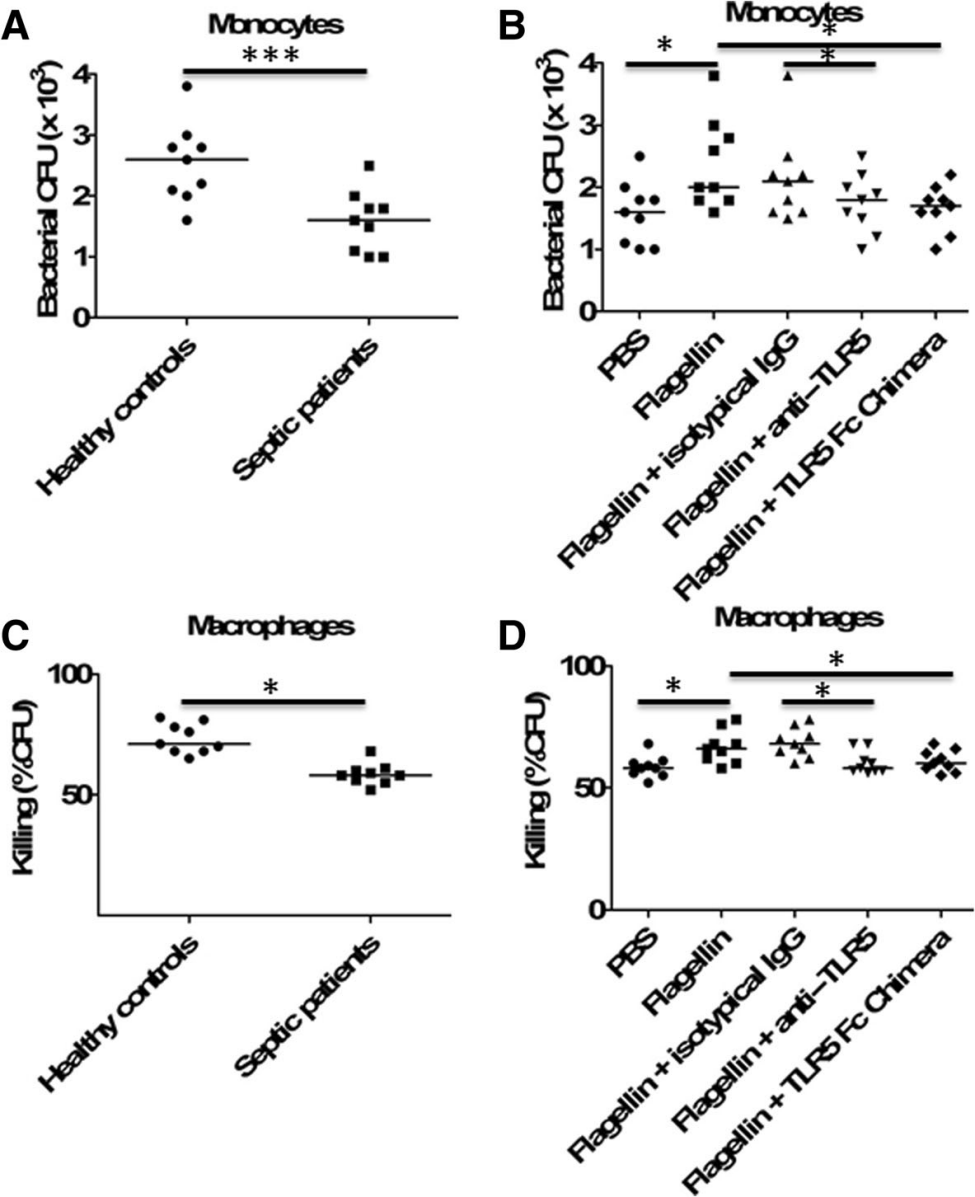
Flagellin treatment improved human monocyte/macrophage bacterial phagocytosis and killing. **a** Monocytes from healthy donors (*n* = 9) and patients who died of sepsis (*n* = 9) were infected with *E. coli* (multiplicity of infection, 10), and phagocytosis function was assessed. ***p < 0.001 when compared between groups (denoted by the horizontal bracket; Mann–Whitney *U* test). **b** Monocytes from healthy donors (*n* = 9) and patients who died of sepsis (*n* = 9) were pretreated with flagellin (100 ng/ml) in the presence or absence of anti-TLR5 antibodies (1 μg/ml) or TLR5 Fc Chimera (1 μg/ml) for 24 h, and then infected with *E. coli* (multiplicity of infection, 10). At the indicated times, phagocytosis function was assessed as described. *p < 0.05 when compared between groups (denoted by the horizontal bracket; Mann–Whitney *U* test). **c** Monocyte-derived macrophages (MDM) from healthy donors (*n* = 9) and patients who died of sepsis (*n* = 9) were infected with *E. coli* (multiplicity of infection, 10), and bacterial killing function was assessed. *p < 0.05 when compared between groups (denoted by the horizontal bracket; Mann–Whitney *U* test). **d** Monocyte-derived macrophages (MDM) from healthy donors (*n* = 9) and patients who died of sepsis (*n* = 9) were pretreated with flagellin (100 ng/ml) in the presence or absence of anti-TLR5 antibodies (1 μg/ml) or TLR5 Fc Chimera (1 μg/ml) for 24 h, and then infected with *E*. *coli* (multiplicity of infection, 10). At the indicated times, the bacterial killing function was assessed as described. *p < 0.05 when compared between groups (denoted by the horizontal bracket; Mann–Whitney *U* test)

We further investigated the effects of flagellin on human monocytes/macrophages. Human monocytes were isolated from peripheral blood of septic non-survivors and healthy controls. As expected, HLA-DR, a surrogate marker for immunosuppression in septic patients [14], was significantly decreased on monocytes from patients who died of sepsis when compared with healthy controls (Additional file 1: Figure S4). Furthermore, monocytes from patients who died of sepsis displayed significantly lower phagocytosis of *Escherichia coli* compared with that of monocytes from healthy individuals (Fig. 8a). Flagellin treatment could restore bacterial phagocytosis of monocytes from septic patients, while blocking TLR5 using anti-TLR5-neutralizing antibodies or recombinant TLR5 Fc chimera could suppress the ability of flagellin to enhance bacterial phagocytosis by human septic monocytes (Fig. 8b).

Human monocytes were further differentiated into monocyte-derived macrophages using M-CSF. The bacterial killing ability of macrophages derived from septic non-survivors was significantly lower than that of macrophages derived from healthy controls (Fig. 8c). Flagellin treatment could significantly enhance bacterial killing of macrophages derived from septic patients, while blocking TLR5 using anti-TLR5-neutralizing antibodies or recombinant TLR5 Fc chimera could suppress the ability of flagellin to enhance bacterial killing by human septic macrophages (Fig. 8d).

## Discussion

In the present study, we demonstrated that the administration of flagellin effectively prevented the progression of sepsis in the CLP polymicrobial sepsis model in a macrophage-dependent manner. Flagellin activation of macrophages promoted the protective immunity against bacterial infection and improved survival in polymicrobial sepsis. We also clearly demonstrated that the therapeutic effects of flagellin required TLR5, and TLR5 deletion could abolish the beneficial effects of flagellin on sepsis. Therefore, flagellin acted through TLR5 to elicit antiseptic activity.

A number of studies have demonstrated the effectiveness of flagellin as an adjuvant for controlling infectious diseases [15, 16]. A recent study has demonstrated that pretreatment of the mice with flagellin 4 h before CLP challenge significantly decreased the sepsis-induced lethality [16]. To assess the therapeutic benefits of flagellin administration at a time when patients are more likely to be treated, flagellin was administered 2–8 h after CLP in this study according to our previous work and others [11, 17, 18]. Importantly, the ability of flagellin administration after the onset of sepsis to significantly improve survival in mice suggests that flagellin is an effective rescue therapy. These results are consistent with a previous study which has demonstrated that administering flagellin to antibiotic-treated mice before VRE infection can reduce VRE colonization [9].

We and others have shown that monocytes/macrophages can play a pivotal role in the resolution of sepsis [11–13, 19, 20]. We have demonstrated that progranulin could protect sepsis by promoting macrophage recruitment [11], while others have shown that mast cells aggravated sepsis by inhibiting macrophage phagocytosis [20]. Furthermore, DJ-1, a well-established ROS scavenger, could impair ROS production for bacterial killing by macrophages, and DJ-1-deficient mice had improved bacterial clearance, reduced organ injury, and increased survival in CLP-induced polymicrobial sepsis compared with wide-type mice [21]. In contrast, sphingosine-1-phosphate receptor 3 (S1PR3) signaling was essential for ROS production and phagolysosomal maturation, which mediated bacterial killing in macrophages. Enhancing endogenous S1PR3 activity using a peptide agonist potentiated ROS production and bactericidal function in macrophages, resulting in decreased bacterial burden, less tissue injury, and improved survival rates in multiple models of sepsis [13]. In this study, we found that depleting macrophages could dramatically impair the survival and bacterial clearance of septic mice treated with flagellin, while adoptive transfer of flagellin-activated macrophages could protect mice against lethal sepsis. Furthermore, flagellin could directly enhance phagocytic function by promoting phagosome formation and bacterial killing by increasing ROS production in macrophages. These data indicate that flagellin administration is a viable therapeutic modality in sepsis by upregulating antimicrobial activity in macrophages. However, the improved outcomes after flagellin treatment of sepsis were not mediated by neutrophils. This result is consistent with a report by Lu et al., which showed macrophages, but not neutrophils, mediated the beneficial effect of leukocyte cell-derived chemotaxin 2 (LECT2) on bacterial sepsis [19], but is at odds with a report by Muñoz et al., which showed that neutrophils were required for flagellin-elicited protection against *Streptococcus pneumoniae* lung infection [22]. One potential explanation for this apparent discrepancy may be the different animal models. We used a CLP-induced sepsis model, whereas Muñoz et al. used a *Streptococcus pneumoniae* pneumonia model. Another explanation for this discrepancy is likely due to the use of different routes of flagellin administration. We used a systemic administration route (intraperitoneal injection of flagellin), whereas Muñoz et al. used a locally mucosal administration route (intranasal injection of flagellin). In addition, a recent study has also shown that endotoxin preconditioning could confer renal epithelial protection in various models of sepsis, which was mediated by macrophages [23].

A recent study has shown that flagellin could suppress experimental asthma by generating regulatory T cells in mice [24]. In this lethal CLP model, there was no significant difference in the regulatory T cell population between mice treated with flagellin and PBS control (data not shown). We acknowledge that the contribution of flagellin-mediated effects on regulatory T cells to long-term immunosuppression in sepsis should be investigated using another sublethal model of CLP followed by a secondary infection with external pathogens [25, 26]. For example, IL-33 has been shown to attenuate sepsis by enhancing neutrophil influx to the site of infection in the lethal CLP model [4]. In the sublethal CLP model followed by *Legionella pneumophila* infection, IL-33 also contributed to sepsis-induced long-term immunosuppression by expanding the regulatory T cell population [26]. Furthermore, TLR5 is also expressed on other innate cells and organ epithelium and endothelium cells [6, 7]; the regulatory effects of flagellin on these cells in sepsis should be studied in the future work, which is beyond our present study.

In the development of adjuvant therapies for patients with sepsis, it is imperative that these findings from animal studies should be translated into humans. A recent study has demonstrated that the expression of TLR5 on monocytes on day 1 or 2 could predict SIRS after major abdominal surgery [8]. Here, we further found that TLR5 expression on peripheral monocytes was significantly upregulated in septic patients when compared with healthy individuals. Importantly, we documented for the first time that those septic patients who did not survive had significantly higher expression of TLR5 on circulating monocytes than did the survivors. Our observation that higher expression levels of TLR5 on monocytes were associated with poorer outcomes and an increase in the incidence of bacteremia of septic patients suggests that monocyte TLR5 expression might be a potential indicator of immune dysfunction and mortality. Furthermore, monocytes/macrophages from patients who died of sepsis demonstrated reduced phagocytic activity and bacterial killing ability, while treatment of septic monocytes/macrophages with flagellin restored their phagocytosis and bacterial killing ability as observed in healthy individuals. These data suggest that measuring human monocyte TLR5 expression may guide the use of flagellin to contain the infection and improve survival in septic patients. However, we acknowledge that the clinical value of monocyte TLR5 expression in septic patients should be further validated in a larger size clinical trial.

## Conclusions

In recent years, it has become increasingly clear that immunosuppression plays an important detrimental role in the morbidity and mortality of sepsis [2, 3, 27], and there is a focus on the development of immunostimulatory agents to improve host ability to combat primary infection and reduce secondary infections in sepsis [28, 29]. The current study demonstrated that therapeutic treatment with flagellin could prevent the progression of severe sepsis via TLR5. Importantly, macrophages, but not neutrophils, were necessary for the beneficial effects of flagellin on sepsis. Our study not only established the potential value of the application of flagellin as an immunoadjuvant in treating sepsis, but also provided new insights into targeted therapeutic strategy on the basis of TLR5 expression on circulating monocytes in septic patients.

## Additional file

Additional file 1: Materials and methods. Figure S1. Therapeutic effects of flagellin in *Escherichia coli*–induced sepsis. Flagellin (5 μg) or saline control was injected intraperitoneally into C57BL/6 mice (*n* = 20) at 2 h after intraperitoneal infection of 5 × 10^8^ *Escherichia coli.* (A) Survival of flagellin- or saline-treated mice after *Escherichia coli* infection. Comparison between groups was done by Kaplan–Meier analysis followed by log-rank tests. ***p < 0.001 when compared with mice treated with phosphate-buffered saline (PBS) control. (B) Bacterial counts in PLF or blood from mice (*n* = 5 per group) treated with or without flagellin (5 μg) at 24 h after *Escherichia coli* infection. Horizontal bars represent median values, and dots represent individual mice. ***p < 0.001 when compared between groups (denoted by the horizontal bracket; Mann–Whitney *U* test). Figure S2. Role of flagellin in the production of proinflammatory cytokines and chemokine during sepsis. Flagellin (5 μg) was injected intraperitoneally into CLP-induced septic mice, after which the levels of cytokines and chemokines in the PLF and blood were measured at 48 h after lethal CLP. *p < 0.05, **p < 0.01, ***p < 0.001 when compared between groups (denoted by the horizontal bracket; Mann–Whitney *U* test). Figure S3. Representative flow cytometric analysis of TLR5 expression on the surface of monocytes. Figure S4. HLA-DR expression levels on the surface of circulating monocytes from healthy donors (*n* = 9) and patients who died of sepsis (*n* = 9). Horizontal bars represent median values, and dots represent individual participants. ***p < 0.001, compared between groups (denoted by the horizontal bracket; Mann–Whitney *U* test). Table S1. Characteristics of septic patients, ICU controls and healthy controls. Table S2. Characteristics of sepsis survivor and non-survivor (DOCX 578 kb)

## Abbreviations

ALT: Alanine transaminase
APACHE II: Acute Physiology and Chronic Health Evaluation II
AST: Aspartate transaminase
CFUs: Colony-forming units
CLP: Cecal ligation and puncture
LDH: Lactate dehydrogenase
MDM: Monocyte-derived macrophages
PBMC: Peripheral blood mononuclear cell
ROS: Reactive oxygen species
SOFA: Sequential [Sepsis-related] Organ Failure Assessment
TLRs: Toll-like receptors
