# Supplementary material for: Flagellin attenuates experimental sepsis in a macrophage-dependent manner
Source: Crit Care. 2019 Apr 3;23:106. doi: 10.1186/s13054-019-2408-7 (PMC6446324; doi:10.1186/s13054-019-2408-7)
Supplement: Supplementary file 1 — Materials and methods. Figure S1. Therapeutic effects of flagellin in Escherichia coli–induced sepsis. Flagellin (5 μg) or saline control was injected intraperitoneally into C57BL/6 mice (n = 20) at 2 h after intraperitoneal infection of 5 × 10^8^ Escherichia coli. (A) Survival of flagellin- or saline-treated mice after Escherichia coli infection. Comparison between groups was done by Kaplan–Meier analysis followed by log-rank tests. ***p < 0.001 when compared with mice treated with phosphate-buffered saline (PBS) control. (B) Bacterial counts in PLF or blood from mice (n = 5 per group) treated with or without flagellin (5 μg) at 24 h after Escherichia coli infection. Horizontal bars represent median values, and dots represent individual mice. ***p < 0.001 when compared between groups (denoted by the horizontal bracket; Mann–Whitney U test). Figure S2. Role of flagellin in the production of proinflammatory cytokines and chemokine during sepsis. Flagellin (5 μg) was injected intraperitoneally into CLP-induced septic mice, after which the levels of cytokines and chemokines in the PLF and blood were measured at 48 h after lethal CLP. *p < 0.05, **p < 0.01, ***p < 0.001 when compared between groups (denoted by the horizontal bracket; Mann–Whitney U test). Figure S3. Representative flow cytometric analysis of TLR5 expression on the surface of monocytes. Figure S4. HLA-DR expression levels on the surface of circulating monocytes from healthy donors (n = 9) and patients who died of sepsis (n = 9). Horizontal bars represent median values, and dots represent individual participants. ***p < 0.001, compared between groups (denoted by the horizontal bracket; Mann–Whitney U test). Table S1. Characteristics of septic patients, ICU controls and healthy controls. Table S2. Characteristics of sepsis survivor and non-survivor (DOCX 578 kb) [file 13054_2019_2408_MOESM1_ESM.docx]

Supplementary Materials and methods

**Tissue histology**

Mice were sacrificed at the indicated times after surgery, after which their lungs, livers, kidneys and spleens were fixed, sectioned, and stained with hematoxylin and eosin for morphological analysis.

**Pathology score assessment**

Mice subjected to sham or cecal ligation puncture (CLP) were sacrificed at the indicated times after surgery, after which their lungs, livers, kidneys and spleens were fixed in 4% formalin, and embedded in paraffin. 4-micrometer sections were stained with hematoxylin and eosin (H&E) and analyzed by a pathologist blinded for groups using a BH2 Olympus microscope (Olympus). To score lung inflammation and damage, the entire lung surface was analyzed with respect to the following parameters: bronchitis, edema, interstitial inflammation, intra-alveolar inflammation, pleuritis, endothelialitis and percentage of the lung surface demonstrating confluent inflammatory infiltrate. Each parameter was graded from 0 (absent) to 4 (severe). Livers, kidneys and spleens were scored according to the following parameters: number of thrombi, number of (micro) abscesses, presence and degree of inflammation, and presence and degree of necrosis. Each parameter was graded from 0 (absent) to 3 (severe). The total pathology scores for lungs, livers, kidneys and spleens were expressed as the sum of the score for all parameters.

**Immunohistochemistry for apoptosis evaluation**

Mice were euthanized at 24 hours after surgery, after which their spleens were isolated. The TUNEL (terminal deoxynucleotidyltransferase [TdT]–mediated dUTP nick end labeling) assay was performed with paraffin-embedded tissue sections, which were first deparaffinized according to a standard histological protocol. The sections were then permeabilized with Triton X-100 at 4°C for 2 minutes and flooded with TdT enzyme and digoxigenin-dUTP reaction buffer (TUNEL) reagent for 60 minutes at 37°C. The percentage of apoptotic (TUNEL-positive) cells was evaluated by counting 500 total cells under a light microscope.

**Flow cytometric analysis**

Human residual blood from the routine sample was used for TLR5 determinations by flow cytometry (FC500 Beckman Coulter) within 4 hours after collection. 20 µl of whole blood in EDTA were labelled with 10 µl of antibody mixture, consisting of equal quantities of fluorescein isothiocyanate (FITC)-conjugated mouse anti-human CD14 (BD Pharmingen™, clone M5E), FITC-conjugated mouse anti-human CD16 (BD Pharmingen™, clone 3G8), phycoerythrin (PE)-conjugated TLR5 (Biolegend, clone S16021I) or Peridinin-Chlorophyll-Protein Complex (PerCP) conjugated- human leukocyte antigen (HLA)-DR (BD Pharmingen™, clone G46-6). After 15 min incubation, red blood cells were lysed with VersaLyse™ Lysing Solution (Beckman Coulter) and 10 min later were analyzed using the flow cytometer. Monocytes were defined as forward- and side-scatter plot and CD14 expression. Neutrophils were defined as side-scatter plot height and CD16 bright expression. Expressions of TLR5 or HLA-DR on 10,000 viable cells were then gated and analyzed by flow cytometry as mean fluorescence intensity (MFI), which included both the changes of TLR5 expression on individual cell and the percentage of cells expressing the TLR5.

To determine macrophage polarization, mouse peritoneal cells were characterized accordingly with PE-conjugated rat anti-mouse F4/80 (BD Pharmingen™, clone T45-2342)、FITC-conjugated rat anti-mouse CD11c (BD Pharmingen™, clone HL3), and allophycocyanin (APC)-conjugated rat anti-mouse CD206 (AbD Serotec, clone MR5D3) monoclonal antibodies. At least 10, 000 cells were collected with a FACScan flow cytometer (Becton Dickinson) and analyzed with FC Express software.

**Immunohistochemistry for apoptosis evaluation**

Mice were euthanized at 24 hours after surgery, after which their spleens were isolated. The TUNEL (terminal deoxynucleotidyltransferase [TdT]–mediated dUTP nick end labeling) assay was performed with paraffin-embedded tissue sections, which were first deparaffinized according to a standard histological protocol. The sections were then permeabilized with Triton X-100 at 4°C for 2 minutes and flooded with TdT enzyme and digoxigenin-dUTP reaction buffer (TUNEL) reagent for 60 minutes at 37°C. The percentage of apoptotic (TUNEL-positive) cells was evaluated by counting 500 total cells under a light microscope.

**Quantification of cytokines and chemokines**

Assessment of inflammatory cytokines and chemokines including IL-1β, TNF-α, IL-6, IL-10, CXCL1, and CCL2 was performed by using ELISA kits (Biolegend) according to the manufacturers’ instructions.

**Serum biochemistry**

Blood was collected in tubes with heparin after cardiac puncture, centrifuged. Alanine transaminase (ALT), aspartate transaminase (AST), lactate dehydrogenase (LDH) and creatinine were determined with commercial available kits (Sigma-Aldrich, St. Louis, MO), using a Hitachi analyzer (Boehringer Mannheim, Mannheim, Germany) according to the manufacturers’ instructions.

**Determination of bacterial colony-forming units (CFUs)**

Serial dilutions of peripheral blood or peritoneal lavage fluid of mice were plated on blood-agar plates. The spleens were also isolated, and 10 mg of each tissue was homogenized in 700 μl of PBS. 50 μl of the tissue homogenate was then cultured overnight on blood–agar plates at 37°C. CFUs were determined after 24 h.

**Isolation of murine macrophages and neutrophils**

For isolation of peritoneal macrophages, mice were injected with 5 ml PBS. Macrophages were isolated from peritoneal lavage by plastic adherence. For isolation of neutrophils, mice were injected intraperitoneally (i.p.) with 1 mL 3% thioglycollate broth (Sigma-Aldrich). Elicited cells were harvested 4 h later by peritoneal lavage with 5 ml of cold PBS, neutrophils were then purified by discontinuous Percoll gradient centrifugation followed by magnetic cell sorting (Miltenyi Biotec).

***In vivo* microphage depletion**

The clodronate-encapsulated liposomes and PBS-encapsulated liposomes were firstly prepared. Clodronate was purchased from Roche Diagnostics. Phosphatidylcholine was obtained from Lipoid, and cholesterol was purchased from Sigma-Aldrich. Clodronate-encapsulated liposomes were delivered i.p. (200 μL) to deplete macrophages. PBS-encapsulated liposomes were delivered in a similar fashion as a control. Clodronate liposome treatment resulted in a 90% decrease of macrophages in the spleen by flow cytometry using PE-conjugated anti-F4/80 (eBioscience).

***In vivo* neutrophil depletion**

To deplete neutrophils, mice were injected intravenously (i.v.) with 0.1 mg of RB6-8C5 monoclonal antibodies (mAb) to mouse Ly6G (eBioscience, clone RB6-8C5) with rat IgG2b as a control. This treatment depleted 95% of neutrophils in the blood and 90% of neutrophils in the spleen by flow cytometry using PE-conjugated anti-CD11b (BD pharmingen) and FITC-conjugated Ly6C (BD pharmingen).

**Adoptive transfer of macrophages**

Peritoneal macrophages were treated with 5 μg/ml flagellin or PBS saline for 24 hours to activate macrophages. At 48 hours after macrophage depletion, 1 x 10^9^ macrophages treated with flagellin or PBS saline suspended in 100 μl pyrogen-free PBS were injected i.p. into the mice.

**Isolation of human monocytes and culture of monocyte-derived macrophages (MDM)**

Human monocytes were isolated from peripheral blood collected into Becton Dickinson (Franklin Lakes, NJ) Vacutainer acid citrate dextrose tubes and differentiated into monocyte-derived macrophages (MDM) in 50 ng/ml macrophage colony-stimulating factor (M-CSF; Invitrogen) for 6 to 7 days.

**Reactive oxygen species (ROS) measurement**

Peritoneal macrophages were incubated with heat-inactivated *E. coli* (multiplicity of infection = 20) for 0, 30, 60 or 90 min. To measure the total intracellular ROS levels, macrophages were treated with the fluorogenic probe H2DFFDA (Life Technologies) at 5 μM for 30 min at 37°C. The medium was then removed, and the cells were returned to prewarmed fresh growth medium. The emitted fluorescence was detected by a fluorescent microplate reader using 490/520 nm excitation/emission filters (Molecular Devices, Sunnyvale, CA). The ROS levels are reported as fluorescence intensity.

**Electron microscopy**

Peritoneal macrophages were treated for 24 hours with flagellin and challenged with zymosan (20 particles/macrophage) for 30 minutes at 37°C. For transmission electron microscopy (TEM), macrophages were plated on bacteriologic plastic and fixed with the use of a mixture of 2.5% glutaraldehyde, 2% paraformaldehyde, and 0.1% picric acid in 100mM cacodylate buffer (pH 7.0) containing 2mM EGTA and 1mM MgCl2. The samples were post-fixed in 1% osmium in 100mM cacodylate buffer (pH 7.0) for 1 hour at 4°C, washed with distilled water, and stained en bloc with 2% aqueous uranyl acetate for 2 hours at 4°C, in the dark. The samples were dehydrated with ethanol, and the cells were released from plastic using propylene oxide. The cells were pelleted and washed several times with propylene oxide and embedded in resin. Ultrathin (~70-nm thick) sections were cut, stained with uranyl acetate and lead citrate, and examined in a FEI Tecnai 12 electron microscope.

For scanning electron microscopy, macrophages were plated on coverslips and fixed as described in the paragraph above. Samples were rinsed several times with distilled water, dehydrated through a series of ethanol washes, and critical point-dried. After sputter-coating with gold, samples were examined in a JOEL JSM 5510 scanning electron microscope. Images were viewed with an FEI Tecnai 12 microscope (FEI UK Ltd) operating at 80 kV with a 20-μm objective aperture using a Gatan US1000 1 2k-2k CCD camera and Gatan DigitalMicrograph software Version 3.11.1. Images were then analyzed.

**Phagocytosis assays**

FITC-labeled *E.coli* were prepared by incubation with 0.5 mg/ml FITC (Sigma) for 20 min at 37°C. Macrophages/monocytes (1 x 10^5^ cells) or neutrophils (1 x 10^6^ cells) were incubated with FITC-labeled bacteria at a multiplicity of infection of 100 for 30 min at 37°C. After washing steps, cell nuclei were stained with DAPI (Invitrogen), followed by visualization using confocal laser scanning microscopy (LSM 510, Zeiss). The ratio of engulfed bacteria (as determined by overlay of green bacteria) was quantified by an independent researcher from 300 counted cells per well and was expressed as percentage of cells that contain bacteria.

**Bacterial killing assays**

Human or murine macrophages (1 x 10^5^ cells) were infected with *E.coli* (multiplicity of infection, 10) at 37 °C for 1 h, and they were washed with buffer containing tobramycin (100 µg/ml) to remove extracellular bacteria and were lysed. Live intracellular bacteria were quantified by culture of lysates for determination of bacterial uptake (t = 0 h) and intracellular killing (t = 2 h). Killing was calculated from the percentage of colonies present at t = 2 h as compared to t = 0 h, as follows: 100 − [number of CFUs t = 2 hours/number of CFUs t = 0 hour]. In another experiment, neutrophils (1 x 10^6^ cells) were infected with *E.coli* at an MOI ratio of 1:100 at 37 °C for 30 minutes, and cells were resuspended in medium containing 100 μg/mL tobramycin to remove extracellular bacteria, and then lysed in PBS containing 0.1% Triton 100 for assessment of uptake (t = 0 h), and additional samples were incubated for 1 additional hour (t = 1 h) to assess bacterial killing as described above.

**Supplementary Figures**

**
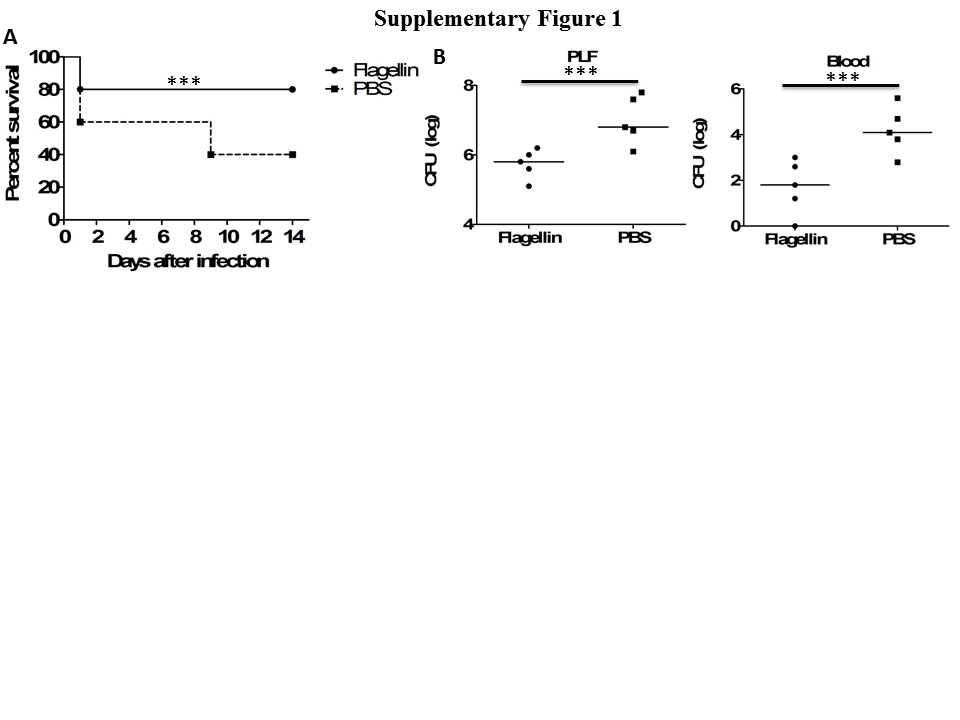
**

**Figure S1. Therapeutic effects of flagellin in *Escherichia coli*-induced sepsis.** Flagellin (5 μg) or saline control was injected intraperitoneally into C57BL/6 mice (n=20) at 2 hours after intraperitoneal infection of 5 x 10^8^ *Escherichia coli.* (A) Survival of flagellin- or saline-treated mice after *Escherichia coli* infection. Comparison between groups was done by Kaplan–Meier analysis followed by log–rank tests. ****p*<0.001 when compared with mice treated with phosphate-buffered saline (PBS) control. (B) Bacterial counts in PLF or blood from mice (n = 5 per group) treated with or without flagellin (5 μg) at 24 hours after *Escherichia coli* infection. Horizontal bars represent median values, and dots represent individual mice. ****p*<0.001 when compared between groups (denoted by horizontal bracket; Mann–Whitney *U* test).


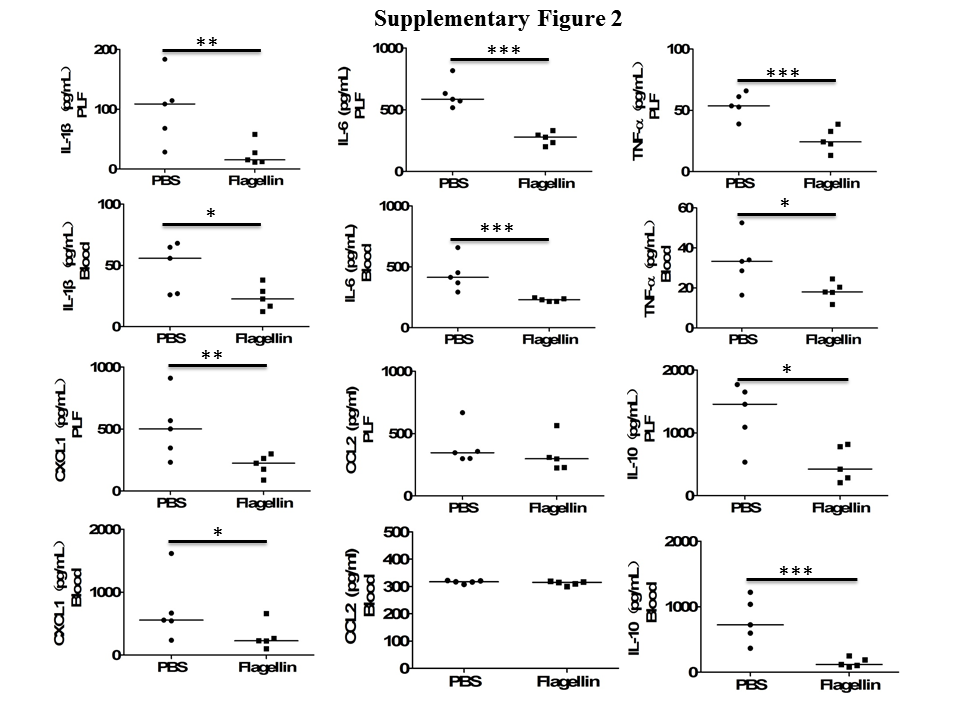


**Figure S2. Role of flagellin in the production of proinflammatory cytokines and chemokine during sepsis.** Flagellin (5 μg) was injected intraperitoneally into CLP–induced septic mice, after which the levels of cytokines and chemokines in the PLF and blood were measured at 48 hours after lethal CLP. **p*<0.05, ***p*<0.01, ****p*<0.001 when compared between groups (denoted by horizontal bracket; Mann–Whitney *U* test).


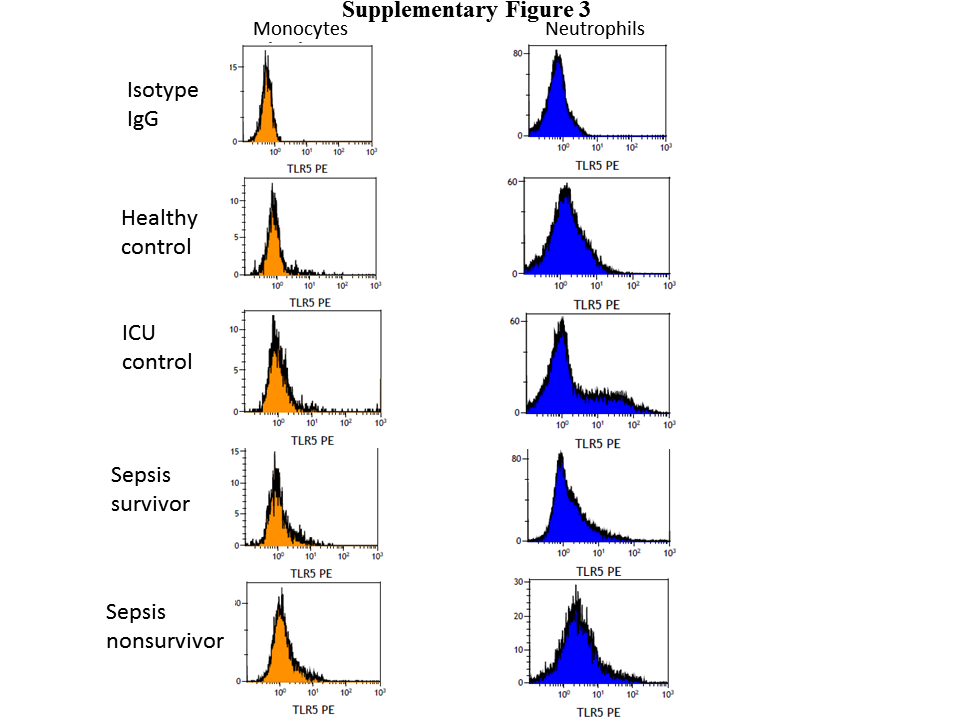


**Figure S3. Representative flow cytometric analysis of TLR5 expression on the surface of monocytes.**

**
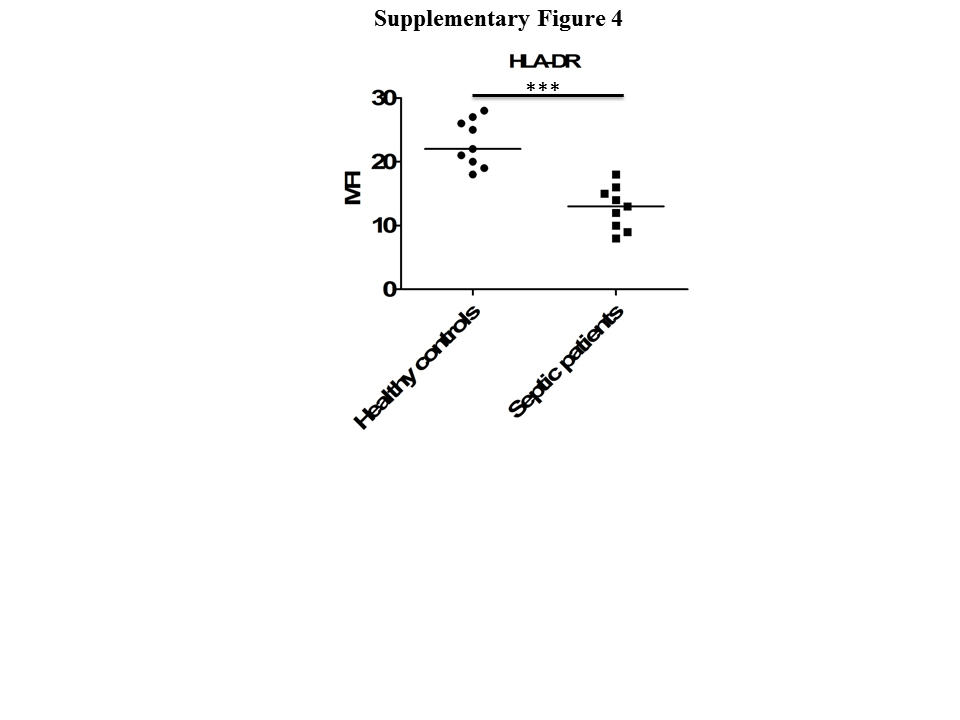
**

**Figure S4.** **HLA-DR expression levels on the surface of circulating monocytes from healthy donors (n=9) and patients who died of sepsis (n=9).** Horizontal bars represent median values, and dots represent individual participants. ****p*<0.001, compared between groups (denoted by horizontal bracket; Mann–Whitney *U* test).

**Table S1 Characteristics of septic patients, ICU controls and healthy controls**

Characteristics Sepsis patients (n=53) ICU controls (n=23) Healthy controls (n=37)

Male sex 39 17 28

Age, years 55 (49–71) 52 (46-70) 51 (45–68)

WBC, 10^9^/L 10 (6-19) 7 (4-9)** 6 (4-9)***

CRP, mg/L 136 (36-261) 5 (0-10)*** NA

Infection site, no. of patients

Respiratory 19 NA NA

Abdominal 11 NA NA

Vascular 5 NA NA

Urinary 3 NA NA

Other 5 NA NA

Bacteremia 33 NA NA

Isolates, no. of patients

Gram positive 21 NA NA

Gram negative 27 NA NA

Miscellaneous 5 NA NA

APACHE II score 17.3 (12.1-25.9) 5.1 (2.1-7.3)*** NA

SOFA score 8.5 (2.8-18.0) 0.6 (0.2-1.7)*** NA

ICU stay, days 8 (3-17) 4 (3-8)** NA

Died/survived 23/30 0 NA

NOTE. Data are expressed as median (interquartile range) unless otherwise indicated. APACHE II: acute physiology and chronic health evaluation II; SOFA: sequential organ failure assessment; ICU: intensive care unit; WBC: white blood cells; CRP: C-reaction protein; NA: not applicable.

***p*<0.01, ****p*<0.001 when compared with sepsis patients (Mann–Whitney *U* test).

**Table S2 Characteristics of sepsis survivor and non-survivor**

Characteristics Survivors (n=30) Non-survivors (n=23)

Male sex 23 16

Age, years 53 (49–71) 57 (50–71)

WBC, 10^9^/L 10 (7-16) 9 (6-19)

CRP, mg/L 140 (40-261) 168 (36-212)

Lactates, mmol/L 3.0 (1.0-5.2) 3.9 (2.1-6.5)*

APACHE II score 15.2 (12.1-19.1) 18.3 (16.7-25.9)*

SOFA score 7.7 (2.8-13.6) 11.2 (6.1-18.0)**

ICU stay, days 8 (3-17) 10 (6-14)

Any hydrocortisone use^#^ 21 17

NOTE. Data are expressed as median (interquartile range) unless otherwise indicated. APACHE II: acute physiology and chronic health evaluation II; SOFA: sequential organ failure assessment; ICU: intensive care unit; WBC: white blood cells; CRP: C-reaction protein.

^#^Use of hydrocortisone or its equivalent (hydrocortisone dose = 4 x prednisolone dose, 5 x methylprednisolone dose, 25 x dexamethasone dose).

**p*<0.05, ***p*<0.01 when compared with survivors (Mann–Whitney *U* test).
